# Critical role of Tim-3 mediated autophagy in chronic stress induced immunosuppression

**DOI:** 10.1186/s13578-019-0275-1

**Published:** 2019-01-22

**Authors:** Anna Qin, Ting Zhong, Huajiao Zou, Xiaoya Wan, Bifeng Yao, Xinbin Zheng, Deling Yin

**Affiliations:** 1 Xiangya School of Pharmaceutical Sciences, Central South University, Changsha, 410008 Hunan China; 2 Department of Internal Medicine, College of Medicine, East Tennessee State University, Johnson City, TN 37614 USA

**Keywords:** Chronic stress, Autophagy, Tim-3, Galectin-9, Immune suppression

## Abstract

**Background:**

Psychological and physical stress can either enhance or suppress immune functions depending on a variety of factors such as duration and severity of stressful situation. Chronic stress exerts a significantly suppressive effect on immune functions. However, the mechanisms responsible for this phenomenon remain to be elucidated. Autophagy plays an essential role in modulating cellular homeostasis and immune responses. However, it is not known yet whether autophagy contributes to chronic stress-induced immunosuppression. T cell immunoglobulin and mucin domain 3 (Tim-3) has shown immune-suppressive effects and obviously positive regulation on cell apoptosis. Tim-3 combines with Tim-3 ligand galectin-9 to modulate apoptosis. However, its impact on autophagy and chronic stress-induced immunosuppression is not yet identified.

**Results:**

We found remarkably higher autophagy level in the spleens of mice that were subjected to chronic restraint stress compared with the control group. We also found that inhibition of autophagy by the autophagy inhibitor 3-methyladenine (3-MA) significantly attenuated chronic stress-induced alterations of pro-inflammatory and anti-inflammatory cytokine levels. We further elucidated that 3-MA dramatically inhibited the reduction of lymphocyte numbers. Moreover, chronic stress dramatically enhanced the expression of Tim-3 and galectin-9. Inhibition of Tim-3 by small interfering RNA against Tim-3 significantly decreased the level of autophagy and immune suppression in isolated primary splenocytes from stressed mice. In addition, α-lactose, a blocker for the interaction of Tim-3 and galectin-9, also decreased the autophagy level and immune suppression.

**Conclusion:**

Chronic stress induces autophagy, resulting with suppression of immune system. Tim-3 and galectin-9 play a crucial regulatory role in chronic stress-induced autophagy. These studies suggest that Tim-3 mediated autophagy may offer a novel therapeutic strategy against the deleterious effects of chronic stress on the immune system.

## Background

Psychological or physical stress can increase or decrease the immune functions in both humans and animals, depending on the severity and duration. We and others have shown that chronic stress could inhibit immune functions and increase susceptibility to diseases [1–5]. Acute stress enhances while chronic stress suppresses cell-mediated immunity in vivo: a potential role for leukocyte trafficking [3–6]. Chronic stress induces a suppressive effect on innate and adaptive immune responses by altering the Type 1–Type 2 cytokine balance and suppressing numbers, trafficking, and function of immunoprotective cells, which will increase susceptibility to stress-related diseases such as infections and cancers [1, 3]. Nonetheless, much work remains to be done to further elucidate the psychological and physical mechanisms by which chronic stress induces immune suppression and weakens health or exacerbates diseases. The mouse model of restraint stress has been widely used by us and others to investigate the effect of stress on the immune system [3, 7–9]. Physical restraint restricts an animal’s movement and access to food and water [9, 10]. In addition to physical immobilization, psychological stress plays a significant part in this model [10].

Autophagy is illustrated as intracellular lysosomal degradation and recycling of proteins and organelles, which has become an essential process in maintaining the homeostasis of humans and animals through modulating the fundamental cellular and organismal metabolism [11, 12]. Autophagy and its machinery have indispensable roles in immunity, including functions in pathogen clearance, thymic selection, antigen presentation, immune cell development and maintenance, and regulation of cytokine production [13–15]. Misregulation of autophagy can result in susceptibility to autoimmune and inflammatory diseases including chronic inflammatory bowel disease, rheumatoid arthritis, multiple sclerosis, systemic lupus erythematosus (SLE), infectious diseases and cancers [14, 16]. With these diverse and extensive immune-related functions for autophagy, it is essential to further explore the role of autophagy in the modulations of immune suppression following chronic stress.

T-cell immunoglobulin and mucin domain 3 (Tim-3, gene name Havcr2) is an immunoglobulin (Ig) and mucin domain family cell-surface molecule, which has been observed to exert immune-suppressive effects in multiple cell types, including effector T cells, regulatory T cells (Tregs), and innate immune cells [17–19]. Experimental results have revealed that the engagement between Tim-3 and its ligands suppresses Th1 and Th17 responses and induces peripheral immune tolerance and blockade of the Tim-3 pathway with specific monoclonal antibodies brings about exacerbated autoimmune responses due to abrogation of tolerance in experimental animal models [20, 21]. These results reveal that Tim-3 plays a negative regulatory role in the immune system, indicating that Tim-3 may be involved in the regulation of chronic stress-induced immunosuppressive processes. Apart from this, Tim-3 binds to its ligand and incites an influx of calcium to the intracellular region of Th1 cells, triggering apoptosis, which results in inhibition of Th1-mediated immune responses [20, 22]. Thus, the effect of Tim-3 on apoptosis has been well documented, but its impact on autophagy remains to be elucidated.

Galectin-9 is a member of galectins, a family of carbohydrate-binding proteins that have been linked to a fundamental function in regulating immune cell homeostasis and inflammation [22–24]. Galectin-9 dominantly distributes in leukocytes that are responsible for innate and acquired immunity, thymocytes, activated endothelial cells and fibroblasts stimulated by IFN [25]. Galectin-9 participates in regulating infections, antimicrobial immunity, autoimmune disorders, allergic responses, cancers, and degenerative diseases [24, 26]. Galectin-9 is the first recognized ligand for Tim-3. Previous studies have revealed that galectin-9-induced apoptosis of Th1 via the Ca^2+^-calpain-caspase-1 pathway is Tim-3-dependent in vitro [17, 27]. More importantly, blockade of the galectin-9-Tim-3 interaction reversed Tim-3-mediated suppression in vitro, including reduced T cell apoptosis and increased pro-inflammatory cytokine production [18]. Although a large number of studies have demonstrated the regulation of apoptosis and immune system by the combination of galectin-9 and Tim-3, the effect of this binding on autophagy and chronic stress induced immunosuppression is not known yet.

## Methods

### Animals and experimental model of restraint stress

BALB/c male mice (6–8-week old) were obtained from the Animal Breeding Center of Central South University. Mice were subjected to an established chronic physical restraint stress [3–5]. Briefly, mice were placed in a 50-ml conical centrifuge tube with multiple punctures to allow ventilation. Mice were held horizontally in the tubes for 12 h. Control littermates were kept in their original cage without food and water for 12 h. After physical restraint, mice were sacrificed and blood samples and spleen specimens were collected for further tests. Animal care was conducted according to the Guide for the Care and Use of Laboratory Animals enacted by the US National Institutes of Health. The study was approved by the Animal Research Committee of Center of Central South University.

### Animal treatment protocol

One hour before the initiation of the physical restraint, the animals received a single intraperitoneal (i.p.) injection of α-lactose (300 mM, 300 μl/20 g body weight; Sigma, Darmstadt, Germany) [28] or autophagy inhibitor 3-methyladenine (3-MA) (20 mM, 300 μl/20 g body weight, Selleck, Houston, USA) [29] in 300 μl of sterile saline or 1 μl of solvent control (DMSO) in 300 μl sterile saline for the controls. After physical restraint, mice were sacrificed and blood samples and spleen specimens were collected for further analyses.

#### CD4^+^ T cell sorting

Splenic naive CD4^+^ T cells were negatively selected by using the MagCellect™ cell selection kits and reagents (R&D Systems, Minneapolis, Minn, USA). Isolated CD4^+^ T cells were collected for further investigation.

### Cell apoptosis detection by flow cytometric analysis

Cell apoptosis was determined by flow cytometric analysis of Annexin V-FITC/PI double-staining. CD4^+^ T cells were harvested according to the manufacturer’s instructions and rinsed with cold PBS twice by centrifugation at 1000*g*, resuspended in 195 μl binding buffer (Beyotime, Shanghai, China). 5 μl Annexin-V (Beyotime, Shanghai, China) and 10 μl PI (Beyotime, Shanghai, China) were then added to the solution, and cells were gently vortexed and incubated for 15 min at room temperature in darkness. Stained cells were analyzed by flow cytometer (Becton, Dickinson and Company, CA, USA).

### Western blot analysis

Western blotting was performed as described previously [30]. Briefly, the cellular proteins were fractionated by 12% SDS-PAGE gel and electroblotted onto Immobilon^®^ PVDF Membranes (Merck KGaA, Darmstadt, Germany). After blocking with nonfat milk, the membranes were blotted overnight at 4 °C with primary antibodies (Table 1). After incubation with HRP-conjugated secondary antibodies (goat anti-rabbit IgG, Proteintech, China), membranes were then visualized with an enhanced chemiluminescent detection kit (Cwbio, Beijing, China).

**Table 1:** Antibodies used for western blotting

Name	Description	Manufacturer
Anti-Beclin 1	Rabbit monoclonal, 60 kDa	Proteintech (11306-1-AP)
Anti-p62	Rabbit monoclonal, 62 kDa	Proteintech (18420-1-AP)
Anti-LC3	Rabbit monoclonal, 14, 16 kDa	CST (#3868)
Anti-Tim-3	Rabbit monoclonal, 33 kDa	Abcam (ab185703)
Anti-galectin-9	Rabbit monoclonal, 45 kDa	Proteintech (17938-1-AP)
Anti-β-actin	Rabbit monoclonal, 43 kDa	Proteintech (20536-1-AP)

### Immunofluorescence

Poly-l-lysine was applied to promote isolated CD4^+^ T cell adhesion to solidsubstrates. Then isolated CD4^+^ T cells were fixed with 4% paraformaldehyde for 30 min at room temperature, permeabilized with anti-CD4 primary antibody (Proteintech, Wuhan, China) overnight at 4 °C and subsequently incubated with secondary antibodies (donkey anti-rabbit IgG, YEASEN, Shanghai, China) at 37 °C for 30 min. Nuclei were stained with DAPI for 10 min and cells were visualized with fluorescence microscope (Nikon, Tokyo, Japan).

### Histopathology and immunohistochemistry (IHC)

Spleens were fixed in 4% buffered formalin and embedded in paraffin. Sections were stained with hematoxylin & eosin (H&E) using standard procedures. IHC was performed with a Diaminobenzidine (DAB) Histochemistry kit (ZSGB-BIO, Beijing, China) according to the manufacturer’s instructions. In brief, after antigen retrieval with 1 mM EDTA (pH 9.0), deparraffinized sections were incubated in 1% Blocking Reagent solution for 1 h at room temperature and then labeled with anti-Beclin 1, anti-p62, anti-LC3, anti-Tim-3, anti-galectin-9 diluted 1:100 in PBS overnight at 4 °C. As a negative control, tissues were also stained with the diluent reagent alone. After washing, sections were incubated with Polymer Helper (ZSGB-BIO, Beijing, China) and subsequently with polyperoxidase-anti-mouse/rabbit IgG (ZSGB-BIO, Beijing, China). Finally, the signal was developed with DAB substrate and counterstained with hematoxylin.

### SiRNA transfection

Small interfering RNA (siRNA) oligonucleotides against Tim-3 and negative control siRNA (NC-Si) were designed and synthesized by the RiboBio Co., Ltd. (Guangzhou, China). Splenocytes were isolated from spleens of stressed or unstressed mice and transfected with a mixture of siRNA using Lipofectamine™ 2000 (Invitrogen, Carlsbad, CA, USA) [31]. 5 μl Lipofectamine 2000 dissolved in 295 μl DMEM and 6 μl NC-Si or Tim-3-Si dissolved in 295 μl DMEM were mixed. And the mixture was added into a 6 well culture plate which already had 1.4 ml DMEM and equal amounts of splenocytes (8 × 10^6^ cells/ml) in each well. After 6 h incubation, the medium was removed and replaced by complete culture medium. After treatment for another 24 h, the knockdown efficiency was determined by Western blot analysis. The siRNA sequences used are as follows:

Tim-3-siRNA: 5′-CCTCCATAATAACAATGGA-3′;

Negative control siRNA: 5′-TTCTCCGAACGTGTCACGT-3′.

### Enzyme linked immunosorbent assay (ELISA)

To determine the serum level of IFN-γ, IL-17, IL-10, IL-4, blood was collected from all experimental and control mice immediately after stress. 700 μl of blood was collected from each mouse by cardiac puncture. Samples were allowed to clot for 2 h at room temperature before centrifugation for 20 min at 2000×*g*. Then serum was removed and stored at − 20 °C for subsequent ELISA assay. To determine the level of IFN-γ, IL-17, IL-10, IL-4 in supernatants, equal amounts of splenocytes (8 × 10^6^ cells/ml) were planted in 6-well plates and the supernatants were harvested after transfected with siRNA. The amount of cytokines (IFN-γ, IL-10, and IL-4) was detected by using a Mouse ELISA kit (Cusabio, Wuhan, China). IL-17 was quantified using an ELISA kit from Boster (Wuhan, China).

### Isolation of RNA and real-time quantitative RT-PCR

Total RNA was isolated from mouse spleens using TRIzol reagent (Cwbio, Beijing, China). Real-time PCR was performed as described previously [3, 4]. Briefly, 1 μg of RNA from each sample was used for reverse transcription and synthesis of cDNA using PrimeScript™ RT reagent Kit (Perfect Real Time) (Takara, Japan). PCR was performed using iTaq™ universal SYBR^®^ Green Supermix (Bio-Rad, Hercules, CA). GAPDH expression was used as internal control. The primer sequences used are listed in Table 2.

**Table 2:** Primers used for qRT-PCR

Gene	Forward primer sequence (5′–3′)	Reverse primer sequence (5′–3′)
Tim-3	TGCAGGAGCAGTCAGGATTC	GCTGCTGGCTGTTGACGTAG
GAPDH	GCGACTTCAACAGCAACTCCC	CACCCTGTTGCTGTAGCCGTA

### Statistical analysis

The results were presented as mean ± SD. The data were analyzed using one-way analysis of variance and Student’s t-test. A value of *P* < 0.05 was considered to be statistically significant.

## Results

### Chronic stress induces autophagy in spleens

**Fig. 1:**
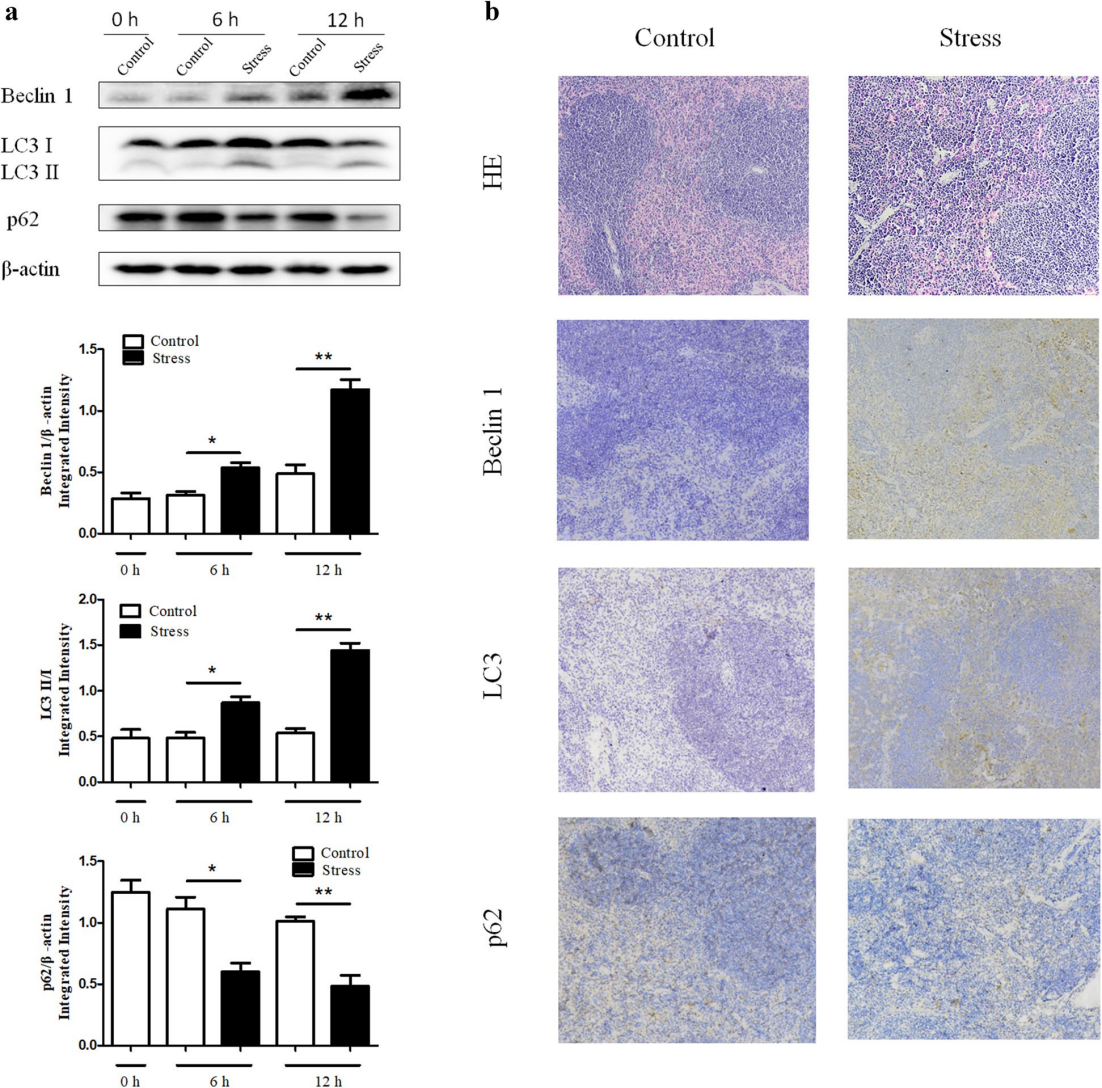
Chronic stress induces autophagy in spleens. We subjected BALB/c male mice aged 6–8-week to physical stress. **a** After stress of the indicated time periods, spleens were harvested. Levels of Beclin 1, LC3, and p62 were determined by Western blot analysis. N = 5 per group. *P < 0.05, **P < 0.01 compared with indicated groups. **b** After 12 h of stress, mouse spleens were harvested and fixed in 4% buffered formalin. Sections were stained with hematoxylin and eosin (100 ×). IHC was performed on adjacent sections with anti-Beclin 1, anti-LC3, anti-p62 Abs and hematoxylin was used as a counterstain (100×). The data are representative of three independent experiments

Growing evidence suggests that autophagy contributes to both the innate and adaptive immune systems [13]. To investigate whether autophagy plays a role in chronic stress-induced immunosuppression, the expression levels of LC3, Beclin 1, and p62 were examined by Western blot analysis and IHC. As shown in Fig. 1a, the activation of Beclin 1 and the conversion from LC3-I to LC3-II were dramatically increased in a time dependent manner following chronic stress. On the other hand, p62, an autophagy adaptor that recruits polyubiquitinated cargo into the autophagy machinery where it also undergoes degradation changed in the opposite way, supporting the rising level of autophagy. Furthermore, spleens from stressed mice exhibited higher autophagy level by IHC than those from control mice (Fig. 1b). Taken together, these results suggest that chronic stress induces autophagy in mouse spleens.

### Chronic stress-induced autophagy is through Tim-3

**Fig. 2:**
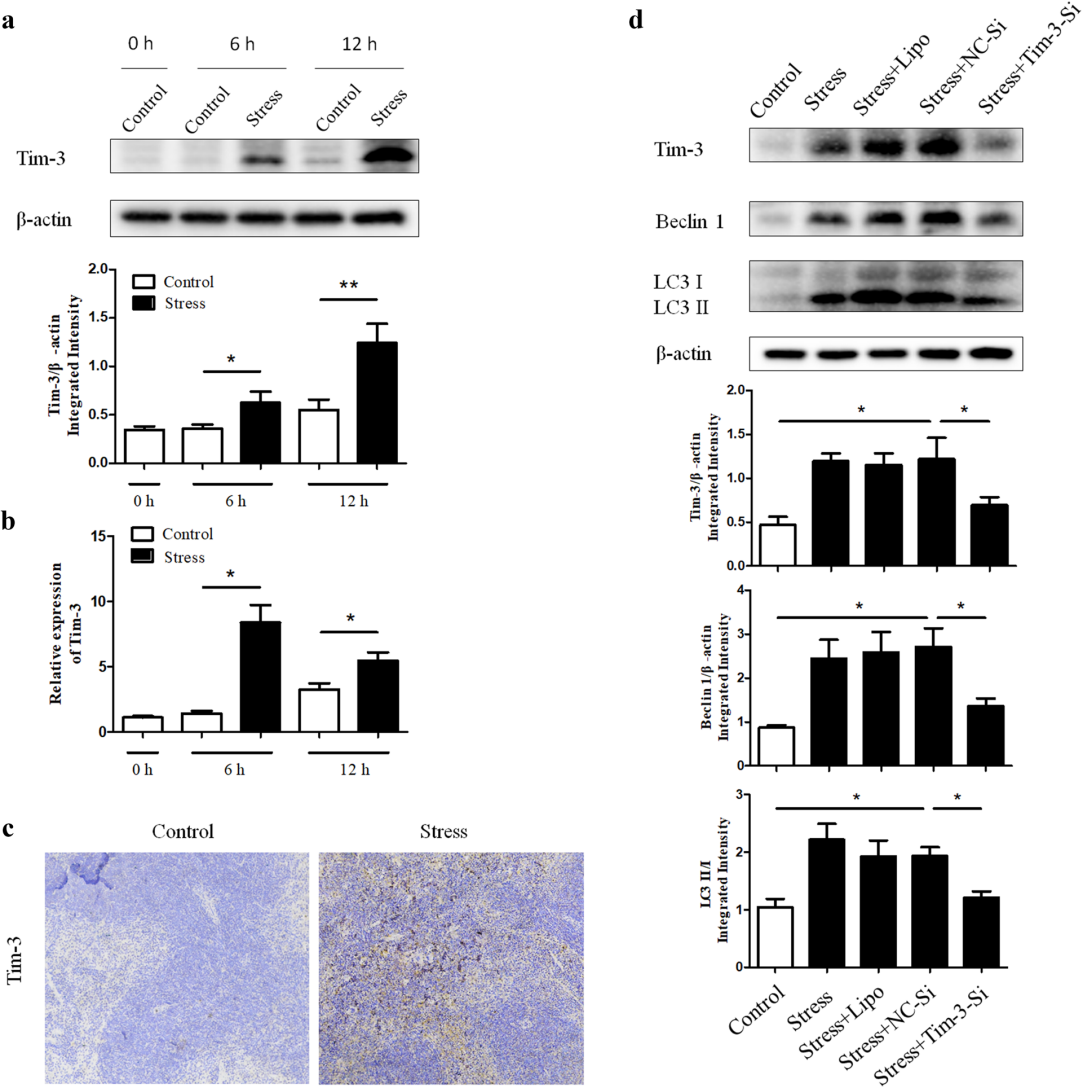
Chronic stress-induced autophagy is through Tim-3. BALB/c male mice (N = 5 per group) were sacrificed after 6 h or 12 h of chronic stress. Cellular lysates were extracted from mouse spleens. **a** The expression of Tim-3 was determined by Western blot. *P < 0.05, **P < 0.01 compared with indicated groups. **b** Total RNA was isolated from mouse spleens, and Tim-3 mRNA levels were determined by quantitative RT-PCR. **c** After 12 h of stress, mouse spleens were harvested and fixed in 4% buffered formalin. IHC was performed on adjacent sections with anti-Tim-3 Ab and hematoxylin was used as a counterstain (100×). **d** The primary splenocytes we isolated from stressed or unstressed mice were transfected with small interfering RNA against Tim-3 (Tim-3-Si) or negative control siRNA (NC-Si) and cellular lysates were obtained, then the Tim-3 and autophagy level were examined by Western blot analysis. *P < 0.05 compared with indicated groups

To determine whether Tim-3 contributes to chronic stress-induced autophagy, we first examined the expression of Tim-3 in spleen tissues of mice following different stress time periods. As shown in Fig. 2a, b, Western blot and qRT-PCR analysis showed the Tim-3 expression in the mouse spleens dramatically increased in a time dependent manner following chronic stress. Consistent with these results, consequence of IHC (Fig. 2c) also manifested the up-regulation of Tim-3 in stressed mice compared with the control group. To assess the requirement of Tim-3 for the autophagic process, small interfering RNA against Tim-3 (Tim-3-Si) was applied in primary splenocytes isolated from stressed or unstressed mice. Interestingly, augmentation of autophagy was obviously diminished by Tim-3-Si employment, which resulted with Tim-3 inhibition (Fig. 2d). These results lead to the suggestion that Tim-3 is required for chronic stress-induced autophagy.

### Stress promotes expression of Tim-3 and autophagy-related proteins in CD4^+^ T Cells

**Fig. 3:**
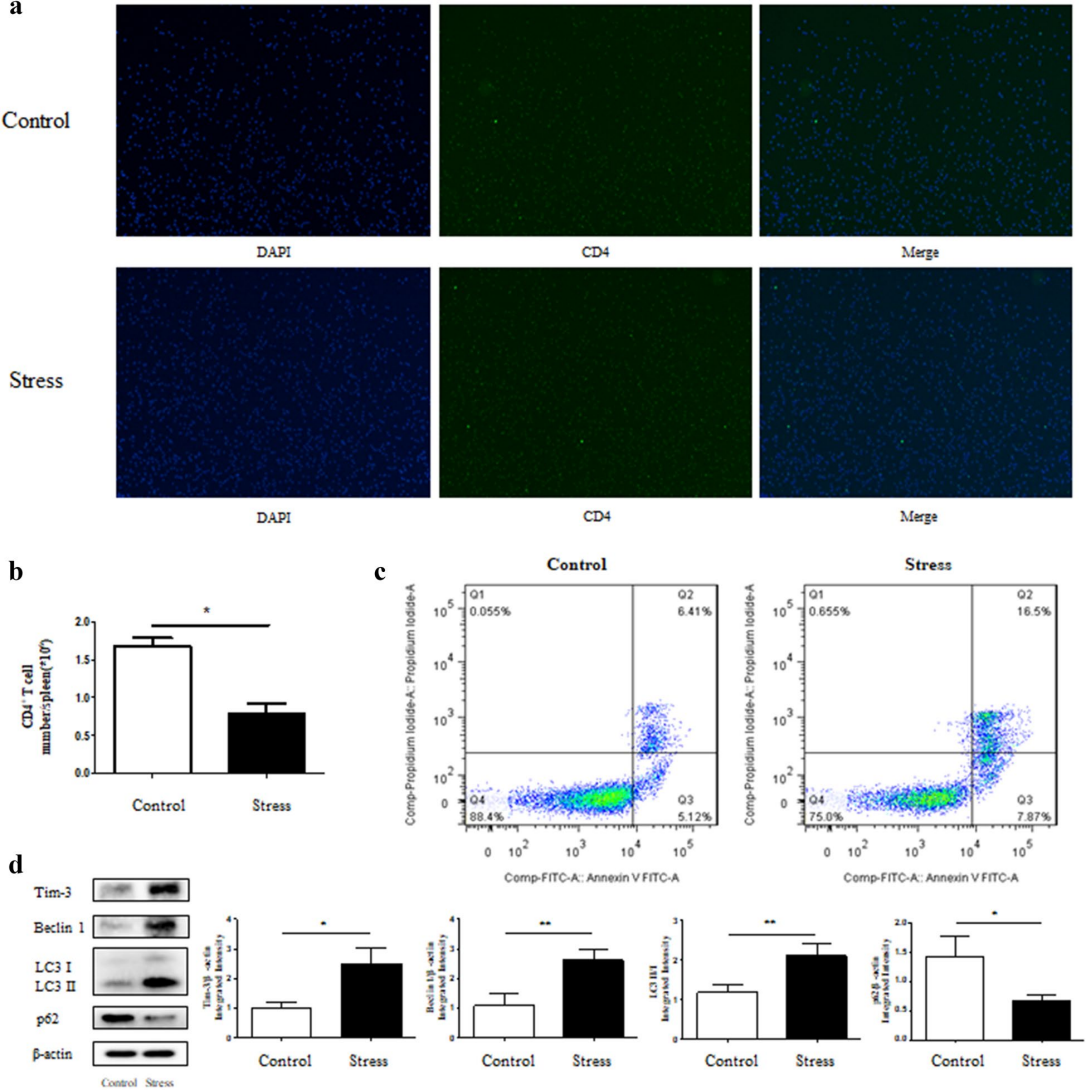
Stress promotes expression of Tim-3 and autophagy-related proteins in CD4^+^ T cell. We obtained CD4^+^ T cells from splenocytes of mice that were subjected to chronic stress for 12 h or unstressed control mice. **a** The purity of resulting recovered CD4^+^ T cells was testified under a fluorescence microscope by immunofluorescence and **b** total splenic CD4^+^ T cells from stressed and unstressed mice were enumerated. **c** Apoptosis of CD4^+^ T cells was examined by Annexin V-FITC and PI staining. **d** Cellular lysates were extracted from the isolated CD4^+^ T cells. The Tim-3 expression and autophagy level were examined by Western blot analysis. N = 3 per group. *P < 0.05, **P < 0.01 compared with indicated groups

CD4^+^ T cells, a fundamental component of splenocytes, can play a part in innate immunity or amplify adaptive immune responses by modifying different cytokine production and functions that determine the changes in the Th1/Th2 cells [32, 33]. Therefore, it is of great significance to explore whether the regulatory function of Tim-3 on autophagy happens in splenic CD4^+^ T cells as well. We first isolated CD4^+^ T cells from splenocytes, and immunofluorescence experiments demonstrated that the CD4^+^ T cells we obtained were of higher purity (> 90% purity) (Fig. 3a). CD4^+^ T cells purified from stressed and control mice were counted and the results showed that restraint stress decreased the number of CD4^+^ T cells (Fig. 3b). To determine whether chronic stress induced lymphocyte reduction was at least in part due to lymphocyte apoptosis, we performed flow cytometry analysis. As shown in Fig. 3c, the detection for cell apoptosis revealed that a much higher apoptotic rate in the stress group comparing with the control group. We also found that the CD4^+^ T cells obtained from the stressed mice expressed significantly higher level of Tim-3 and autophagy-related proteins compared with the control group (Fig. 3d). These results suggest that regulation of Tim-3 on autophagy occurs in CD4^+^ T cells as well as splenocytes.

It has been shown that Tim-3 was expressed in Th1 and Th17, but not in Th2 and may not in Th9 [20, 34]. To determine whether chronic stress alters Tim-3 expression in Tregs, the regulatory T cells were isolated from splenocytes and the expression level of Tim-3 was examined by Western blot analysis. The results showed the regulatory T cells obtained from the mice following 12 h stress expressed significantly higher level of Tim-3 compared with the control group (data not shown).

### Tim-3 mediates immune suppression through modulating autophagy in chronic stress

**Fig. 4:**
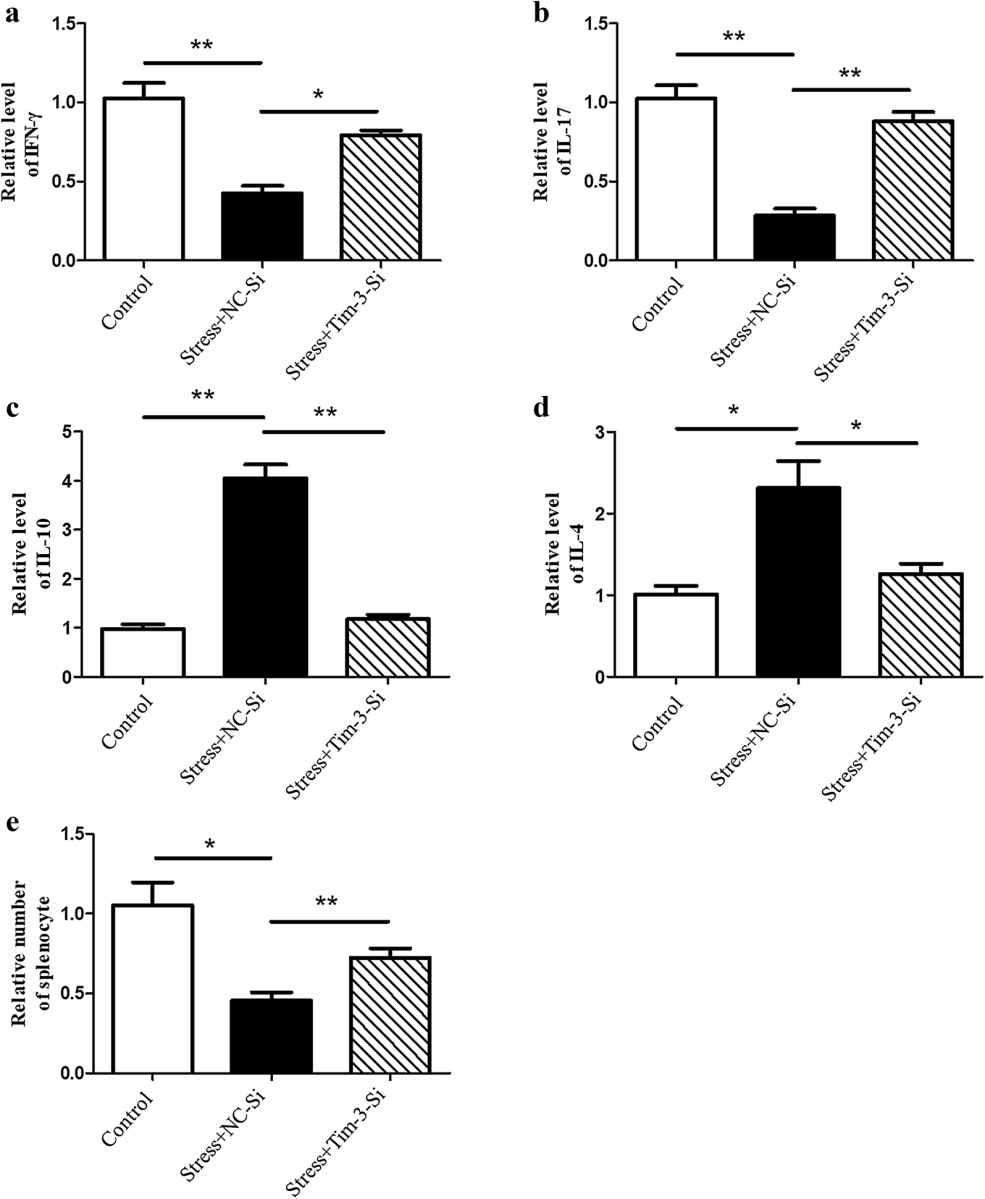
Tim-3 mediates immune suppression following chronic stress through modulating autophagy. The primary splenocytes we isolated from stressed or unstressed mice were transfected with Tim-3-Si or NC-Si and cell supernatants were obtained. The levels of IFN-γ (**a**), IL-17 (**b**), IL-10 (**c**), and IL-4 (**d**) cytokines were detected by ELISA. N = 5 per group. *P < 0.05, **P < 0.01 compared with indicated groups. **e** Total splenocytes were enumerated with a hemocytometer

We have proved chronic stress induces autophagy through Tim-3 (Fig. 2d). We next determined whether the modulation of Tim-3 on autophagy would affect chronic stress-induced immunosuppression. We treated isolated primary splenocytes from stressed and control mice with Tim-3-Si or NC-Si, and then measured Th1 and Th2 cytokine production in cell supernatant. Our results showed that inhibition of Tim-3 by Tim-3-Si rescued the decrease in IFN-γ (Fig. 4a) and interleukin 17 (IL-17) level (Fig. 4b) and decreased IL-10 (Fig. 4c) and IL-4 (Fig. 4d) production in cell supernatant of stressed mice. Importantly, inhibition of Tim-3 significantly diminished the lymphocyte reduction induced by chronic stress (Fig. 4e). Collectively, our data suggests that down-regulation of Tim-3 can rescue the immune suppression following chronic stress through regulating autophagy.

### Galectin-9 regulates autophagy through acting as a ligand for Tim-3

**Fig. 5:**
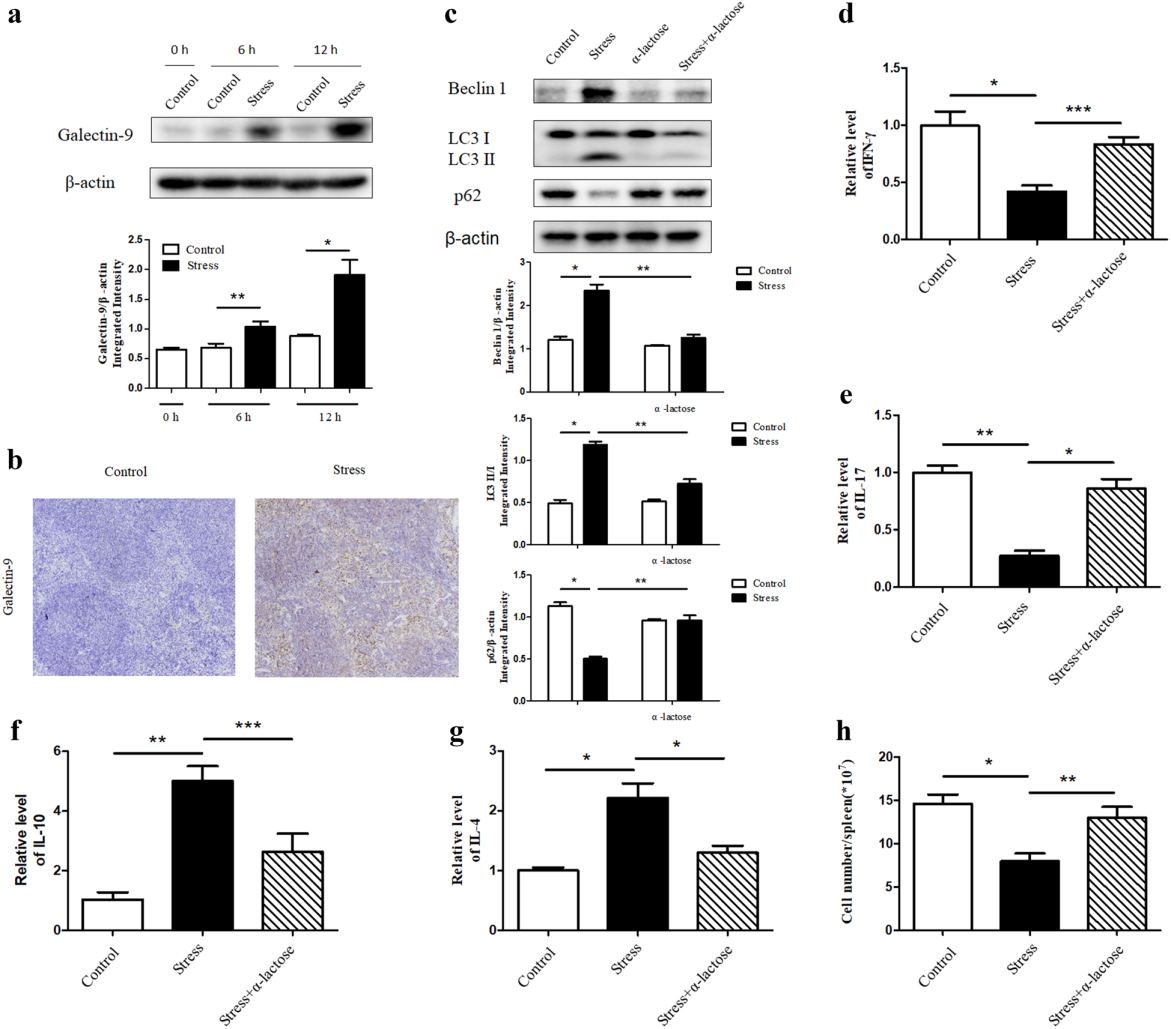
Galectin-9 regulates autophagy through acting as a ligand for Tim-3. **a** BALB/c male mice (N = 5 per group) were sacrificed after 6 h or 12 h of chronic stress. Cellular lysates were extracted from mouse spleens. Expression of galectin-9 was determined by Western blot. *P < 0.05, **P < 0.01 compared with indicated groups. **b** After 12 h of stress, mouse spleens were harvested and fixed in 4% buffered formalin. IHC was performed on adjacent sections with anti-galectin-9 Ab and hematoxylin was used as a counterstain (100 ×). Mice were pretreated with α-lactose. After 12 h chronic stress, the blood and spleens of mice were harvested. **c** Cellular lysates were extracted from mouse spleens. Level of autophagy was determined by Western blot. *P < 0.05, **P < 0.01 compared with indicated groups. The circulating levels of IFN-γ (**d**), IL-17 (**e**), IL-10 (**f**), and IL-4 (**g**) cytokines were examined by ELISA. N = 5 per group. *P < 0.05, **P < 0.01, ***P < 0.001 compared with indicated groups. **h** Total splenocytes were enumerated with a hemocytometer

Galectin-9 is a crucial interaction partner of Tim-3 and is involved actively at various phases of immune response for normal functioning [17, 22, 26]. To clarify whether galectin-9 engages in regulation of autophagy in spleen tissues and in turn influences the immune suppression during chronic stress, the level of galectin-9 in the spleen tissues at different time points was determined by Western blot analysis and showed a growing tendency over stress time (Fig. 5a). The dramatically increased expression of galectin-9 in stressed mice compared with control group was further confirmed by IHC analysis (Fig. 5b). Subsequently, we pretreated mice with α-lactose, a blocker for the combination between galectin-9 and Tim-3 [28, 35], 1 h before the initiation of stress. Interestingly, application of α-lactose could alleviate the stress-induced autophagy in spleens (Fig. 5c). In addition, α-lactose significantly changed the imbalance of cytokines (Fig. 5d–g) and lymphocyte reduction induced by chronic stress (Fig. 5h). Collectively, galectin-9 executes an indispensable role in modulating stress-induced autophagy and immune suppression by functioning as a ligand for Tim-3.

### Down-regulating autophagy restores the stress-induced immune suppression

**Fig. 6:**
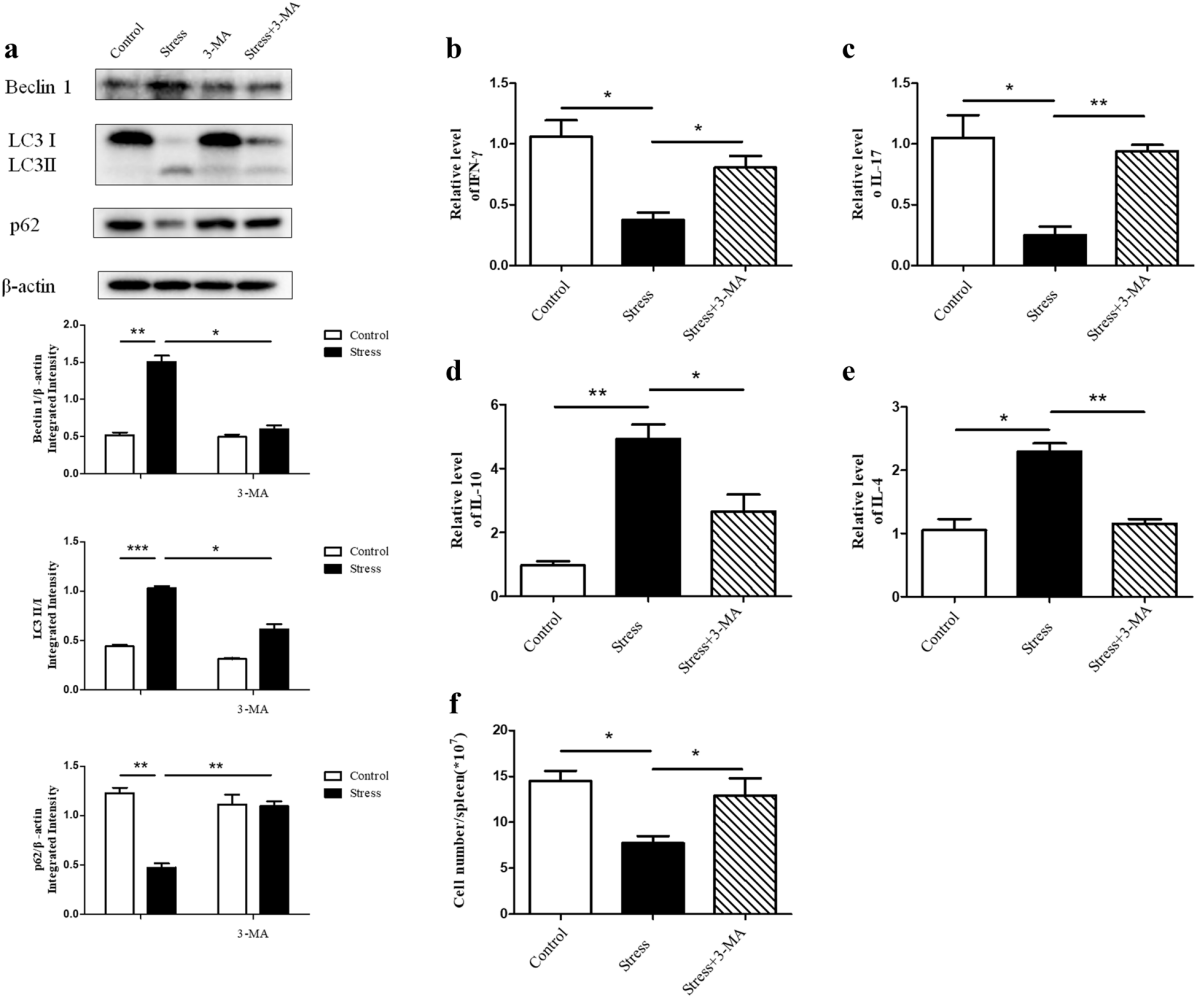
Down-regulating autophagy restores the stress-induced immune suppression. BALB/c male mice were pretreated with 3-MA. After 12 h chronic stress, the blood and spleens of mice were harvested. **a** Cellular lysates were extracted from mouse spleens. Level of autophagy was determined by Western blot. β-actin is shown as a loading control. *P < 0.05, **P < 0.01, ***P < 0.001 compared with indicated groups. The circulating levels of IFN-γ (**b**), IL-17 (**c**), IL-10 (**d**), and IL-4 (**e**) cytokines were detected by ELISA. N = 5 per group. *P < 0.05, **P < 0.01 compared with indicated groups. **f** Total splenocytes were enumerated with a hemocytometer

We have shown that chronic stress induced autophagy in spleen tissues (Fig. 1a, b). We then determined the impact of autophagy on the immunosuppression caused by stress. BALB/c mice were treated with or without the autophagy inhibitor 3-MA 1 h before initiation of restraint stress. As shown in Fig. 6a, the expression of Beclin-1 and the conversion from LC3-I to LC3-II were markedly decreased in stressed mice that were pretreated with 3-MA in parallel with increased level of p62, indicating that 3-MA was effective in suppressing autophagy. We observed the loss of autophagy could reverse the disequilibrium of pro-inflammatory and anti-inflammatory cytokines in serum (Fig. 6b–e). Furthermore, 3-MA was also capable of making the reduction of lymphocyte number caused by chronic stress rebound (Fig. 6f). These data imply that inhibition of autophagy can rescue the immune suppression following chronic stress.

## Discussion

It is well established that various stress model systems of physical stress can either enhance or inhibit immune function depending on the type and duration of the stressors [3–6, 36]. Chronic stress has been verified to suppress immune response [2–6]. In spite of this, the mechanisms responsible for the regulatory effect still remain to be identified. In current study, we firstly find a persistent increase in autophagy level in spleens of mice following chronic stress. Additionally, our findings suggest a critical role of Tim-3 in regulating autophagy in chronic stress and galectin-9 is involved in this process through functioning as a ligand for Tim-3.

Accumulating evidence suggests that autophagy has emerged as a fundamental process for maintaining cellular homeostasis in response to environmental stress and regulates innate and adaptive immunity affecting the pathological outcomes of immune responses [11, 12, 37]. Our results displayed that the level of autophagy in stressed mouse spleens was significantly higher compared with the normal counterparts, nonetheless, application of 3-MA, which could suppress autophagy efficiently, inhibited the reduction of lymphocyte and the changes of Th1/Th2 cytokine secretion in response to chronic stress. Taken together, autophagy is involved in the regulation of chronic stress-induced immunosuppression. Previous studies have disclosed the fact that chronic stress induced lymphocyte reduction, which could be mediated by two possible mechanisms: emigration or cell death [5, 36]. We have discovered the lymphocyte reduction was at least in part due to lymphocyte apoptosis, a prototypical kind of cell death [36]. Historically, the classifications of cell death based on the morphological criteria are apoptosis, necrosis, and autophagic cell death [38]. It has been observed that excessive or uncontrolled levels of autophagy can decrease cell survival through autophagic cell death [14]. We suspect that excessive autophagy following chronic stress could contribute to splenocyte reduction through autophagic cell death and thus result in suppression of immune responses.

Tim-3, identified as immune checkpoint receptor, has been proven to contribute to immune homeostasis by regulating both innate immunity and adaptive immunity [17, 18, 21]. Notably, previous studies revealed Tim-3 down-regulated Th1 responses by transducing apoptotic signaling through galectin-9 engagement [21]. Currently, there is no evidence to clarify the role of Tim-3 in chronic stress-induced immunosuppression and its modulations of autophagy. Our study found the expression of Tim-3 was enhanced both at transcriptional and protein levels in stressed mice in a time-dependent manner. Additionally, the activation of Beclin 1 and conversion from LC3-I to LC3-II, two pivotal indicators of autophagy, were significantly reversed as Tim-3 expression was diminished by the application of small interfering RNA against Tim-3 in isolated primary splenocytes, which indicates that chronic stress induces splenocyte autophagy through Tim-3. It was of great importance that the resulting attenuation of autophagy through Tim-3 knockdown restored splenocyte reduction and disequilibrium of Th1/Th2 cytokine balance, the two significant consequences of chronic stress. Therefore, we can draw the conclusion that Tim-3 plays an immensely significant role in chronic stress-induced immunosuppression through regulating autophagy.

To our surprise, p62, whose expression would increase as the level of autophagy declined in our scenario, was also downregulated after inhibiting the expression of Tim-3 (data not shown). It has been widely accepted that numerous autophagy proteins have multiple functions beyond its canonical role in autophagy, including p62 [39]. For example, it acts as a multifunctional signaling hub by utilizing its different conserved structural elements, which allows p62 to directly interact with protein adaptors at signaling nodes of pathways controlling inflammation, cell death, survival, and metabolic reprogramming [40]. We hypothesize that p62 may impart a function that is autophagy-independent and this function is intimately linked with Tim-3, thus we need further experiments to verify this phenomenon.

Naive CD4^+^ T cells are able to differentiate to Th1, Th2, Th9, Th17, T follicular helper (Tfh) and even induce regulatory T cells to regulate innate immunity or amplify adaptive immune responses [32, 33]. It has been proved the optimal cytokine production of Th1/Th2, the two distinct subsets of CD4^+^ T cells, is crucial in the occurrence and progression of T cells-derived immune responses and their imbalance is blamed for the initiation and development of immune-mediated disease [41]. Our studies displayed that isolated splenic CD4^+^ T cells from the stressed mice augmented the expression of Tim-3 and autophagy-related proteins compared with unstressed control mice. Thus, we suggest chronic stress-induced high expression of Tim-3 significantly elevates autophagy level in CD4^+^ T cells, which results cell death of CD4^+^ T cells and in turn contributes to imbalance of cytokines and immune-mediated disorders.

The observation that Tim-3 expression was increased in chronic stress forced us to focus on galectin-9, the first reported ligand for Tim-3, which has been demonstrated to bind to Tim-3 and trigger a series of events like calcium mobilization, calpain and caspase-1 activation that eventually result in the apoptosis of T cells [22, 27]. Recent results have shown the molecular adaptor human leukocyte antigen B (HLA-B)-associated transcript 3 (Bat3) impedes the Tim-3/Gal-9-mediated apoptosis. The process that Tim-3 ligates galectin-9 is hampered by binding of Bat3 to the intracellular tail of Tim-3 [42, 43]. However, there have been no evidence to clarify whether this combination would affect autophagy so far. Western blot and IHC analysis in the current study display a time dependent increase of galectin-9 expression in stressed mice, suggesting that galectin-9 is activated in response to chronic stress. What’s more, α-lactose pretreatment resulted in the inhibition of stress-induced autophagy, indicating that stress-induced autophagy is modulated by the interaction between Tim-3 and galectin-9. The disequilibrium of Th1/Th2 cytokine balance and reduction of the lymphocyte number caused by chronic stress were rescued after the administration of α-lactose. Taken together, we propose that chronic stress-induced high level of galectin-9 binds to Tim-3 to regulate autophagy and immune system function and this combination could represent a novel and potential therapeutic approach in chronic stress induced immunosuppression.

## Conclusion

In summary, to the best of our knowledge this study is the first to find high level autophagy in spleen tissues of stressed mice. Our results suggest the excessive autophagy plays a role in chronic stress-induced immune suppression. We also provide a novel underlying mechanism that Tim-3 and galectin-9 combine and regulate autophagy and immunosuppression following chronic stress cooperatively. Our studies suggest that further identification of autophagy and the Tim-3-mediated signaling in response to chronic stress may open a new horizon in the prevention and/or treatment of stress induced-immune suppression and infectious diseases.

## Abbreviations

Tim-3: T-cell immunoglobulin and mucin domain 3
3-MA: 3-methyladenine
SiRNA: small interfering RNA
SLE: systemic lupus erythematosus
Tregs: regulatory T cells
IFN: interferon
IHC: immunohistochemistry
H&E: hematoxylin and eosin
NC-Si: negative control siRNA
ELISA: enzyme linked immunosorbent assay
qRT-PCR: real-time quantitative PCR
Tfh cells: T follicular helper cells
